# Integrating Thyroid Hormone Signaling in Hypothalamic Control of Metabolism: Crosstalk Between Nuclear Receptors

**DOI:** 10.3390/ijms19072017

**Published:** 2018-07-11

**Authors:** Soumaya Kouidhi, Marie-Stéphanie Clerget-Froidevaux

**Affiliations:** 1 Laboratory BVBGR, LR11ES31, Higher Institute of Biotechnology of SidiThabet (ISBST), Department of Biotechnology, University of Manouba, Sidi Thabet 2020, Tunisia; soumayakouidhi@gmail.com; 2 Laboratory of Genetics, Immunology and Human Pathology, Faculty of Sciences of Tunis, Department of Biology, University Tunis El Manar, Tunis 2020, Tunisia; 3 CNRS UMR 7221 “Evolution of Endocrine Regulations” Department of “Life Adaptations” MuséumNational d’HistoireNaturelle 57, rue Cuvier CP 32, 75231 Paris, CEDEX 05, France

**Keywords:** energy balance, hypothalamus, thyroid hormone signaling, nuclear receptors

## Abstract

The obesity epidemic is well recognized as a significant global health issue. A better understanding of the energy homeostasis mechanisms could help to identify promising anti-obesity therapeutic strategies. It is well established that the hypothalamus plays a pivotal role governing energy balance. The hypothalamus consists of tightly interconnected and specialized neurons that permit the sensing and integration of several peripheral inputs, including metabolic and hormonal signals for an appropriate physiological response. Current evidence shows that thyroid hormones (THs) constitute one of the key endocrine factors governing the regulation and the integration of metabolic homeostasis at the hypothalamic level. THs modulate numerous genes involved in the central control of metabolism, as *TRH* (Thyrotropin-Releasing Hormone) and *MC4R* (Melanocortin 4 Receptor). THs act through their interaction with thyroid hormone receptors (TRs). Interestingly, TH signaling, especially regarding metabolic regulations, involves TRs crosstalk with other metabolically linked nuclear receptors (NRs) including PPAR (Peroxisome proliferator-activated receptor) and LXR (Liver X receptor). In this review, we will summarize current knowledge on the important role of THs integration of metabolic pathways in the central regulation of metabolism. Particularly, we will shed light on the crosstalk between TRs and other NRs in controlling energy homeostasis. This could be an important track for the development of attractive therapeutic compounds.

## 1. Introduction

According to the World Health Organization (WHO, available online: http://www.who.int/gho/ncd/risk_factors/obesity_text/en/), obesity is no longer viewed as simply a major health problem giving rise to a global “obesity epidemic”. Indeed, obesity has increased worldwide over recent years and represents a major contributor to morbidity and mortality [1,2], which increases healthcare costs. The metabolic diseases associated with obesity, such as type 2 diabetes mellitus and cardiovascular diseases, are closely linked to perturbations in lipid and glucose metabolism [3,4]. However, the mechanisms controlling the major regulatory pathways are not yet fully understood. Thus, it is interesting to further decrypt the processes that underlie energy homeostasis, as their dysregulations can promote the metabolic diseases described above by unbalancing energy intake and energy expenditure could lead to overweight, and thus, to the comorbidities of obesity.

Obesity has long been considered the result of a long-term disproportion between energy intake and energy expenditure. However, the regulation of metabolism is an intricate process coordinated by the central nervous system (CNS) where specialized neurons control and integrate peripheral signals including nutrient and hormone signals, such as insulin and leptin, to control energy balance [5,6]. These complex biological programs could be also influenced by multiple factors, including environmental, genetic, and epigenetic mechanisms. The hypothalamus is the key brain center controlling feeding behavior, and whose dysfunction is thereby involved in the energy imbalance and its subsequent metabolic disorders [7]. The hypothalamus resides in the medial basal region of the brain. It encloses neurons of the arcuate nucleus (ARC), tightly interconnected with other hypothalamic centers such as the paraventricular nucleus (PVN), the lateral hypothalamic area (LHA), the dorsomedial nucleus (DMN), and the ventromedial nucleus (VMN). These specialized hypothalamic nuclei are able to sense and to integrate diverse nutrients and hormone signals resulting in a change in the expression, secretion and activity of specific neurotransmitters and neuromodulators [8]. As a consequence, energy intake and expenditure are modulated [9].

As a key driver of metabolism, increasing evidence highlights the important role played by thyroid hormones (THs) in the hypothalamus, acting centrally to regulate food intake and energy expenditure [10]. Indeed, several studies have reported that thyroid dysfunction correlates with alterations in energy balance and body weight [11,12], but the exact mechanisms and interactions of the various TH signaling pathways by which the metabolism is integrated and modulated in the brain are still not fully understood. In this review we will examine the current knowledge on the roles played by THs on the regulation of energy balance at the central level, focusing on the interaction among the related metabolic pathways and the nuclear receptor crosstalk.

## 2. Overview of CNS Control of Energy Homeostasis

**Figure 1:**
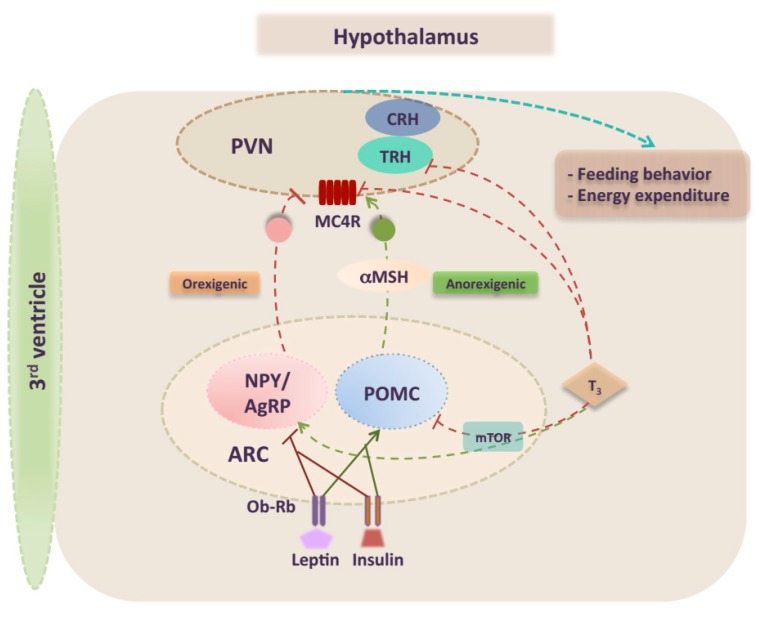
Schematic illustration of hypothalamic regulation of energy homeostasis. Peripheral signals such as leptin and insulin enter the CNS, and act on their specific receptors in key hypothalamic regions that regulate food intake and energy expenditure. Leptin stimulates POMC neurons and inhibits NPY/AgRP neurons in the ARC, resulting in the inhibition of food intake via the action of MC4R-expressing neurons in the PVN, and other brain areas. TH also regulates a number of other metabolic processes by acting on hypothalamic metabolic sensors. Central T3 regulates feeding through mTOR signaling pathway targeting orexigenic and anorexigenic neurons in the ARC, and exerts a negative feedback on TRH and MC4R expression in the PVN. αMSH: melanocyte stimulating hormone; ARC: arcuate nucleus; MC4R: melanocortin 4 receptor; mTOR: mammalian target of rapamycin; Ob-Rb: leptin receptor; NPY: neuropeptide Y; POMC: proopiomelanocortin; PVN: paraventricular nucleus; T3: triiodothyronine; TRH: Thyrotropin-releasing hormone; CRH: corticotropin-releasing hormone; green arrow: activation; red blind-ended arrow: inhibition; solid line: direct action; dashed line: indirect action or other pathway intervention.

During the last decades, a significant number of review articles elegantly summarized the knowledge on the mechanisms and signaling pathways underlying the regulation of energy balance. Even a small dysregulation in one of these pathways can lead to obesity [9]. The hypothalamus is a key brain area that plays a pivotal role integrating whole-body signals and controlling food intake and energy expenditure [13,14]. The hypothalamus is organized into well-structured nuclei [15]. In particular, ARC is the best-characterized nucleus for the regulation of feeding. The ARC has a privileged location in the brain that allows it to tightly sense several signals from the periphery [8,16]. Specifically, in the ARC, there are two well-characterized antagonistic neuronal subpopulations with opposite effects. The NPY–AgRP neurons express orexigenic neuropeptides, agouti-related peptide (AgRP), neuropeptide Y (NPY), and the inhibitory neurotransmitter γ-aminobutyric acid (GABA) [17,18]. POMC neurons express the anorexigenic neuropeptides α-melanocyte-stimulating hormone (α-MSH), a proteolytic product of pro-opiomelanocortin (POMC), and cocaine- and amphetamine-regulated transcript (CART) [19]. Both NPY–AgRP and POMC neurons exert their antagonistic effects by projection to second-order neurons mainly in the PVN (Figure 1), but also in other hypothalamic nuclei (i.e., DMN, LHA, VMN) to modulate feeding behavior. It is well recognized that upon nutrient ingestion α-MSH acts on second order neurons located in the PVN and activates melanocortin 3 (MC3R) and melanocortin 4 (MC4R) receptors, which reduce energy intake and induce energy expenditure [20]. Of note, the PVN neurons display the highest MC4R expression within the hypothalamic area and ligand modulation of MC4R signaling in the PVN profoundly affects feeding. Taken together, the melanocortin pathway is considered as a major anorexigenic circuit in the brain [21,22]. Consistent with this idea, human and mice studies showed that deletion of POMC neurons or their peptide product as well as MC4R deficiency results in obesity [23,24].

Both NPY–AgRP and POMC neurons are directly targeted by circulating hormones such as insulin and leptin (Figure 1) [25,26]. It is well established that leptin, a satiety hormone, plays a key role in the central regulation of energy homeostasis. Leptin is an adipokine encoded by the *LEP* gene, liberated in the circulation by the adipose tissue proportionally to the whole-body fat content and acting as an afferent satiety signal [27]. A significant attention has been given to leptin signaling in ARC, where NPY–AgRP and POMC neurons are the main targets of leptin action via their expression of high levels of leptin receptors (LEPRs) [28,29]. Leptin directly stimulates POMC neurons and activates melanocortin–receptors, while inhibiting the activity of AgRP neurons. Thus, the net effect of leptin signaling within the hypothalamus is increased energy expenditure and reduced body weight. Although it has been believed that melanocortin signaling is a distal mediator of the leptin pathway, there is now evidence that leptin and melanocortin signaling are independent [30]. Emphasizing the importance of leptin, it has been shown that chronic administration of leptin into the brain reduced caloric intake, body weight, and improved glucose sensing in high fat-fed animals [31,32]. Conversely, deficiency of leptin or the ablation of *LEPR* in the CNS induces a morbid obese phenotype [33,34]. Hence, over-reactivation of the LEPR in hypothalamic neurons, including POMC neurons, may induce obesity in high-fat fed mice [35].

## 3. Effects of TH on Central Metabolism

For more than a century, THs have been recognized through clinical observations and experimental studies as a main regulator of the metabolic rate of the whole organism [12]. Actually, THs profoundly affect key metabolic pathways that control energy [36,37]. Thus, it becomes evident that dysregulation of TH levels markedly impacts body weight and metabolism homeostasis in Human [38,39]. Reduced energy expenditure leading to weight gain is observed in hypothyroid patients. Conversely, hyperthyroidism (excess of thyroid hormone), promotes a hypermetabolic state characterized by increased energy expenditure and weight loss [40]. These beneficial effects of TH have led to the development of selective thyroid hormone mimetics as powerful new tools against atherosclerosis and obesity [41,42]. Moreover, the role of TH has been demonstrated particularly in glucose homeostasis [43]. Indeed, hyperthyroid rats showed reduced glucose tolerance and reduced insulin-secretory capacity of β cells, in addition to an increased hepatic insulin resistance [44]. Similarly, hypothyroidism promotes glucose intolerance in hypothyroid non-diabetic mice [45]. These observations are clinically relevant given the increased prevalence of diabetes mellitus in both hypo- and hyperthyroidism [46].

At the peripheral level, TH has been characterized for a long time as directly affecting metabolic tissues (such as liver, white and brown adipose tissue (WAT and BAT), heart, skeletal muscle, …) and controlling the bulk of physiological processes implicated in the modulation of energy expenditure [47,48]. However nowadays, it is well established that THs also promote whole body metabolism and modulate food intake by acting at the central level, in the hypothalamus [49].

TH plasma levels are maintained at the appropriate level to preserve energy homeostasis. This adjustment is due to the integration of a range of metabolic pathways at the hypothalamic level. TH is secreted from the thyroid gland under a flexible and dynamic regulation of the hypothalamic-pituitary axis (HPT) [50,51]. Under normal physiological conditions, the intact HPT axis maintains stable serum TH levels, resulting in a steady TH contribution to energy homeostasis. In the PVN, TRH-producing neurons are sensitively affected by changes in circulating TH. In turn, they define the set point of thyroid gland function by regulating pituitary thyroid-stimulating hormone (TSH) secretion and thus the circulating levels of TH [52,53,54]. Importantly, The TRH-TSH-TH feedback loop is mainly mediated by a direct activation of TR isoform β-dependent signaling to decrease TRH and TSH secretion [55].

Given its crucial role in metabolism regulation that affects all the tissues in the body, TH availability and signaling are tightly controlled by several mechanisms [37,52]. Two THs are derived from the thyroid gland: Thyroxine (T4), the most abundant form and Triiodothyronine (T3), the transcriptionally active form. The TH cellular availability is modulated by enzymes known as deiodinases. Local activation of T4 to the active form T3 by the type 2 5-deiodinase (D2), constitutes a key mechanism of the TH regulation of metabolism. D2 is both expressed at the peripheral and central levels [56,57]. Alongside D2, trans-membrane transporters and TH receptors also control TH signaling pathways, by regulating respectively the TH entrance to the cells and its transcriptional action [58]. Nevertheless, integration of TH signaling occurs both peripherally, in liver, white fat, and BAT, and centrally, in the hypothalamus [11,47].

In addition to mediating the feedback mechanisms regulating THs levels, hypothalamic TH signaling also regulates energy homeostasis by influencing appetite [10,59]. Recent evidence indicates that both orexigenic and anorexigenic neurons are targets of the TH feedback loop in the ARC (Figure 1) [60,61]. It has been shown that centrally-mediated actions of T3 dampens anorexigenic signals by inhibiting POMC mRNA expression. In the fasted state, T3 signaling increases uncoupling protein 2 (UCP2) levels in the hypothalamus thereby stimulating orexigenic pathways by increasing NPY and AgRP levels [62,63]. In the PVN, the T3-induced AgRP release suppresses *TRH* mRNA expression by inhibiting MC4R, a key relay in leptin signaling [64]. This mechanism alters the setpoint of the HPT axis and severely disrupts feeding circuit homeostasis. Recent data have elucidated this link between TH and energy balance, reporting a particular TH-modulation of melanocortin pathway in the hypothalamus [65,66]. Decherf et al. have demonstrated in mice that thyroid status was associated with change of *Mc4r* expression in the hypothalamus. Both qPCR and in situ hybridization showed hypothyroidism to increase endogenous *Mc4r* expression in the PVN, whereas hyperthyroidism repressed *Mc4r* expression in the ARC [65]. Clear evidence from mutagenesis and ChIP assays suggests that T3 can mediate repression of *Mc4r* levels by direct binding of TR to its responsive elements (TREs) on the *Mc4r* promoter. Interestingly, in vivo knockdown or over-expression assays and use of TR isoform-specific knock-in mouse models showed that both TRα and TRβ isoforms play a key role in the *Mc4r* regulation. Thus, the thyroid may be regulated through another negative feedback loop, where MC4R stimulates TH release, which in turn down-regulates *MC4R* expression [65,67,68]. A first physiological relevance of *MC4R* repression caused by T3-negative feedback is an induced weight gain in hypothyroid mice treated with T4. Altogether, these results consolidate the important role of thyroid hormone to tightly drive metabolism in a key energy-related brain area.

It is important to highlight the fact that the central effects of THs are interrelated with master energy sensors in the brain such as AMP-activated protein kinase (AMPK) and mammalian target of rapamycin (mTOR) [69,70]. Over the past few years many studies have focused on AMPK and mTOR pathways that act as global regulators of cellular metabolism in both central nervous system and peripheral organs [71,72]. Specifically, both AMPK and mTOR coexist and interact in the same specific hypothalamic nuclei to respond to nutrient availability and hormonal milieu, regulating energy homeostasis. Cross-regulation between these two signaling pathways is thought to be modulated by TH. It has been shown that hyperthyroidism or central administration of T3 reduces hypothalamic AMPK phosphorylation [73]. In turn, this may upregulate the thermogenic program in BAT and increase weight-gain. In contrast, the hyperthyroid state activates the hypothalamic mTOR signaling pathway associated with upregulation of orexigenic peptides AgRP and NPY in the ARC and increases feeding (Figure1). Interestingly, specific treatment with mTOR inhibitor reversed hyperthyroidism-induced increase in food intake. Notably, it has been suggested that THs directly regulate mTOR in the ARC since it is highly co-expressed with thyroid hormone receptor-α (TRα) in this brain region [74].

## 4. Nuclear Receptors, Integrators of Metabolic Regulation

NRs constitute a major target for hormones and metabolites, which makes them fundamental players in the most important biological process, as metabolism regulation. Indeed, NRs are key metabolic sensors that properly integrate environment changes and energy homeostasis [75,76,77]. Ultimately, a dysfunction of that intricate machinery leads to obesity and type 2-diabetes. Given this important role of NRs, they become increasingly interesting therapeutic targets for various metabolic diseases. In this context, we will particularly focus on: Thyroid hormone receptors (TRs), Nuclear receptors partners (RXR), and nuclear receptor coregulators (PPAR and LXR).

### 4.1. Thyroid Hormone Receptors

At the cellular level, TH transcriptional regulation of metabolic target genes is achieved through hormone-responsive nuclear transcription factors, TRs. *NR1A1* and *NR1A2* are two different genes coding for TRs, which are alternatively spliced to generate four main isoforms: respectively TRα1, TRα2 (which is unable to bind T3) for *NR1A1* and TRβ1and TRβ2 for *NR1A2*. The TR isoforms exhibit varying expression levels both developmentally and spatially within TH target tissues, suggesting a specific tissue-dependent role for each TR isoform. TR transcriptional regulation is modulated by ligand interactions [36,78]. Indeed, a series of highly controlled intracellular processes occurs to ultimately allow for TH binding to its receptors and lead to TH induced target gene transcriptional regulations. These processes include TH transport into the cells, activation or inactivation by deiodinases, and differential expression levels of TR isoforms, nuclear corepressors and coactivators [36,79]. TRs bind to target genes on TREs mainly as functional heterodimeric complexes notably with retinoid X receptors (RXRs) [36,78]. Many studies analyzing TREs sequences in the promoters of T3 target genes have shown that such DNA response elements consist of a core consensus sequence of the hexanucleotide “half-site” (A/G)GGT(C/A/G)A, existing generally in pairs. Such TREs are qualified as positive TREs (pTRE) or negative TREs (nTRE) [79].

At the brain level, all TR isoforms are highly coexpressed throughout the brain, especially in areas related to energy balance such as the ARC, the PVN and the VMN hypothalamic nuclei. TH is the master of HPT axis control by regulating hypothalamic *TRH* gene expression and production in a classical negative feedback loop [80,81]. All the functional TRs are colocalized in the T3-responsive TRH neurons in the PVN [82,83]. Indeed, TRs play isoform-specific roles in T3-dependent repression of *Trh* gene [84]. Specifically, TRβ isoforms play important differential roles in the regulation of *Trh* gene. Both TRβ isoforms bind to the *Trh* promoter at an unusual TRE as a functional heterodimer complex with RXR [85,86], but they induce isoform-specific differential transcriptional regulations.

Three separate nTRE half-sites were characterized in the *Trh* proximal promoter (site 4 from −55 to −60 base pairs (bp); site 5 from +14 to +19 bp; and site 6 from +37 to +42 bp), all of them acting in combination to allow T3-dependent negative regulations [87,88]. Among these nTREs, the *Trh* promoter site 4 appears to be the most important for TH-induced regulation, as it transduces both T3-independent transcriptional activation and T3-dependent repression [89]. Moreover, unlike the other sites, the *Trh* site 4 preferentially binds TR/RXR heterodimers [90].

Likewise, TH directly represses *Mc4r* in the hypothalamus. The T3-induced *Mc4r* repression is achieved via a putative nTRE (half-site), the TRE1, a non-classical sequence identified in the *Mc4r* promoter, different to *Trh* site 4, and thought to bind monomeric nuclear receptors to mediate their transcriptional activities [65]. According to Chip results, *Mc4r* TRE1 recruited high levels of TRβ in presence of T3, whereas low levels were recruited in absence of T3. Indeed, overexpression of TRβ1 or TRβ2 enhanced the repressive effects of T3 on *Mc4r* transcription, independently of the isoforms. Furthermore, the same inhibitory TRβ mediated effect was observed in newborn and adult mice. Besides, TRα isoform is also involved in *Mc4r* regulation. Overexpression of TRα reduced T3-independent transcription. This result was confirmed in TRα knockout mice *TRα °*/*°*, where *Mc4r* expression was increased [65].

Collectively, extensive studies demonstrate that TRs are major physiological regulators of food intake and energy homeostasis, not only peripherally, but also centrally through their action regulating HPT axis. It is well documented that mice and humans with negative mutations of TRα and TRβ display a variety of metabolic phenotypes of induced weight gain and reduced energy expenditure. Consistent with these observations, recent data showed that selective *TRβ* knockdown specifically in the VMN, a key brain region for the control of energy homeostasis, results in marked obesity similarly to murine models with the most extreme forms of monogenic obesity [91]. Furthermore, in vivo studies showed that activation of the TRβ isoform with selective agonists increased the metabolic rate and prevents glucose intolerance, hyper-triglyceridaemia and body weight gain in obese or high-fat diet fed rats [92]. Moreover, mutant mice with specific *TRα* mutation developed visceral adiposity and insulin resistance [93]. Finally, it has been advanced that targeting TR subtypes improve global metabolic outcomes [94].

### 4.2. Retinoid X Receptor (RXR)

Molecular endocrinology has known great progress after the characterization of orphan receptors, in particular, the RXR. Indeed, the discovery of RXR and its ligand led to stupendous concepts in the nuclear receptor research area [95,96]. Mainly, this important finding reveals a not yet defined signaling pathway, which can be modulated by specific ligands. Furthermore, it led to define the interconnection of multiple signaling pathways, especially by the discovery of RXR heterodimerization with different nuclear receptors [97,98]. Three RXR subtypes were identified: RXRα, RXRβ and RXRγ, each encoded by different genes [99]. The three isoforms are highly conserved and share the same mechanisms of heterodimerization with partners. To mediate transcriptional regulation, RXR binds as homodimer, to a direct repeat of half-sites separated by one nucleotide (i.e., a DR1 element) in response to RXR ligands, which might be 9-cis retinoic acid (9cRA). Because of the unique nucleotide in the spacer between the twoDR1 half-sites, it seems that the DR1 sequence underwent Evolution to generate novel binding motifs (DR2, DR3, DR4, etc.). This flexibility of RXR allows it to adopt multiple conformations and thereby dimerize with different nuclear receptors [97,99]. Thus, RXR could play dual roles in NR signaling.

Interestingly, RXRs are involved in several metabolic pathways because of their heterodimerization with nuclear receptor partners, where they can modulate transcription through ligand activation. Hence, the modulation of transcription could be achieved either by the RXR ligand or the partner receptor ligand. This kind of dual-ligand regulation resulted into two categories of heterodimers: permissive and non-permissive [100]. Permissive receptor partners like PPARs, LXRs and FXR are those that can be activated either by ligands of RXR or its partner, and the presence of both ligands results in a cooperative response [101,102]. Non-permissive partners like TRs, VDRs and RARs, are those that can be only activated by the partner’s ligand and function primarily as hormone receptors, while RXR is silent [103,104].

An example of RXR in regulating metabolic pathway is its strong heterodimerization with TR, increasing thereby TR binding to the TRE and amplifying its transcriptional activity [105]. Indeed, it has been shown that RXR increases stimulatory TR responses of TH target genes [106,107]. Moreover, RXR/TR heterodimers play roles in both basal transactivation and T3 suppression of negatively regulated genes. Nevertheless, the role of RXRs in T3-dependent repression showed more complexity than T3-dependent activation. In a context of T3 negative regulation, in vitro results showed that RXR subtypes improve T3 dependent *Trh* regulation, independently of their DNA-binding properties. Likewise, RXRs increase the dominant negative effect of some mutant TRs on specific nTREs [108]. The activation of endogenous RXR by specific ligands increased *Tsh* mRNA levels [109] but did not show any effect on *Trh* expression or preproTRH levels [110]. A functional study demonstrates that knockdown and overexpression of RXRα and RXRβ change hypothalamic *Trh* levels, suggesting differential roles of both RXR subtypes to modulate T3-dependent *Trh* transcription [111].

### 4.3. Peroxisome Proliferator-Activated Receptors (PPARs)

PPARs are nuclear transcription factors belonging to the steroid receptor superfamily. They regulate target genes by binding to peroxisome proliferator hormone response elements (PPREs), generally as a heterodimer with RXR [112]. There are three known PPAR isoforms, PPARα, PPARγ and PPARδ, differentially expressed among tissues. PPARα is abundant in the liver, brown adipose tissue, heart, and kidney; PPARγ is mainly enriched in the adipose tissue and PPARβ/δis ubiquitously expressed throughout the body [113]. PPARs act as fundamental players in various physiological and pathological processes, especially in energy metabolism. Particularly, PPARα and PPARγ isoforms have been extensively documented mainly because they are activated by clinically-used molecules [114]. PPARα is described as a master regulator of lipid metabolism as it controls genes involved in hepatic fatty acid oxidation. PPARα is increasingly described as a potential molecular target of the hypolipidemic drugs for the treatment of dyslipidemia and fibrates. Its exogenous activation decreases both circulating triglyceride levels and reduces lipid stores in liver, muscle, and adipose tissue [115,116]. PPARγ is highly expressed in adipose tissue and plays key roles in adipogenesis, promoting the expression of specific adipocyte markers such as adipocyte lipid binding protein (aP2), phosphoenolpyruvate carboxylase (PEPCK) or lipoprotein lipase (LPL) [117,118]. Moreover, PPARγ is also involved in fatty acid uptake and storage and in glucose metabolism in many other peripheral tissues [119]. Interestingly, PPARγ is the target of thiazolidinedione (TZD), the only current class of insulin-sensitizing drugs in patients with type 2 diabetes [120]. However, several side effects are caused by long-term use of these drugs (mainly an increase of weight gain in both patients and rodent models). Finally, the less-described PPAR isotype, PPARδ, appears as an attractive therapeutic target in metabolic syndrome. PPARδ is ubiquitously expressed and is involved in fatty acid catabolism, energy uncoupling in adipose tissue and muscle, insulin sensitizing, and the reduction of inflammation [121].

Recent evidence supports a new potential role for PPARs in central energy homeostasis regulation. In the CNS, all PPAR subtypes, PPARα, PPARδ and PPARγ, have been involved in the regulation of energy homeostasis. Particularly, PPARγ seems to play a key role in the regulation of energy balance. New studies suggest that exogenous activation of central PPARγ by its ligand TZD leads to weight gain which may contribute to obesity [122,123,124].

Consistently, hypothalamic activation of central PPARγ by either specific agonists or overexpression, leads to enhance positive energy balance. However, inhibition of PPARγ activity in the brain with antagonists or by shRNA-mediated knockdown results in negative energy balance [123]. Interestingly, specific inhibition of PPARγ in the CNS improves the sensitivity of the leptin pathway in the hypothalamus of high-fat diet-(HFD)-fed animals. Recently, a model of transgenic mice lacking PPAR**γ** in POMC neurons showed increased energy expenditure, while body weight and food intake were reduced. Furthermore, these models showed improved glucose metabolism when exposed to HFD [124]. Besides, peripheral administration of either a PPARγ activator or inhibitor failed to affect food intake of mice with POMC-specific *Pparγ* ablation. Taken together, PPARγ signaling in the brain seems to profoundly impact energy balance and to promote the obesity phenotype [125]. The same obesogenic effects have been demonstrated for activation of PPARα in the brain. Conversely, PPARδ seems to play inverse role than PPAR**γ**. In mutant mice lacking *Pparδ* via genetic deletion, *Pparγ* and *Pparα* are highly expressed in the hypothalamus which would potentiate diet induced obesity [126].

### 4.4. Liver X Receptors (LXRs)

Dysregulated cholesterol levels and metabolism represent hallmarks of diseases such as diabetes and atherosclerosis. The LXRs are members of the nuclear receptor family and are considered as major sensors of cholesterol and lipid homeostasis in mammals [127]. Two related LXRs isoforms have been identified: LXRα (encoded by *NR1H3*) and LXRβ (encoded by *NR1H2*), which are a part of the emerging significant newer drug targets within the NR family [128]. Both LXRs isoforms are structurally similar and are activated by the same ligands, however, their tissue expression differs. LXRα is highly expressed in metabolically active tissues and cell types such as liver, intestine, adipose and macrophages, whereas LXRβ is expressed ubiquitously. These transcription factors, activated by cholesterol metabolites, control the expression of a panel of genes involved in cellular cholesterol traffic in a tissue-dependent manner, thereby protecting the cell from cholesterol overload [129,130]. In addition to their central role in cholesterol metabolism, LXRs are key regulators of lipogenesis and have an impact on systemic glucose homeostasis [128]. Thus, inhibiting hepatic LXR seems unlikely to be a successful therapeutic strategy for type 2 diabetes.

Both LXR isoforms are expressed in the brain [131]. However, their central functions are not as well understood as their roles in peripheral organs such as the liver. Neurons need a tightly controlled cholesterol rate regulation for an appropriate synaptic functioning. There is evidence to support that LXRs in the brain regulate pathways that maintain cholesterol balance and activate anti-inflammatory pathways [132]. In fact, studies in isolated murine neurons and glial cells have generally confirmed the ability of LXRs to regulate the expression of genes linked to cholesterol transport, including *ABCA1* and *APOE24*. Thus, the brain LXRs signaling exhibits neuroprotective mechanisms and anti-inflammatory effects [133]. However, a perturbation of such pathway increased both cellular cholesterol and amyloid-β levels in the brain. Such deregulation constitutes a fundamental mechanism in the development of Alzheimer’s disease. Thereby, a particular link between LXR signaling and Alzheimer’s disease has been established [134].

Recent data indicate that LXR could modulate set-points of the HPT axis andMC4R pathways in the hypothalamus [135]. Thus, activated LXR represses TRH levels and induces the orexigenic peptides, which may promote weight gain and obesity. In contrast, specific inactivation of LXRs enhances *Trh* expression in the hypothalamus [135] and induces the browning of WAT, thereby ameliorating obesity outcomes [136,137].

## 5. TR Crosstalk with PPAR and LXR to Regulate Metabolism

**Figure 2:**
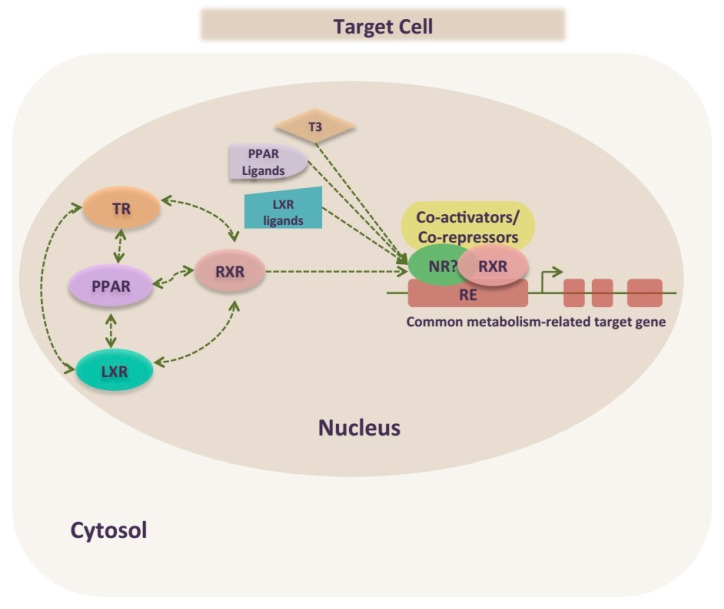
Nuclear receptors crosstalk targeting metabolic pathways. Thyroid hormone signaling involves TR crosstalk with other nuclear hormone receptor including PPAR and LXR, for the transcriptional control of metabolic gene expression. Although NRs interaction is an intricate mechanism that needs further investigations, it could be explained at least by the competition to bind similar DNA response elements (RE) on common metabolism-related target genes and to form heterodimers with RXR that exists in limiting amounts. Also, another interaction could be a reciprocal effect on their expression. Dashed line: direct or indirect crosstalk between signaling pathways.

It is well established that the thyroid hormone is a key factor regulating basal metabolic rate, thermogenesis, glucose metabolism, lipolysis, and HPT axis. Increasing data have focused on specific actions of TH in metabolic regulations. These include the molecular mechanisms of TRs actions on cholesterol and carbohydrate metabolism, through direct actions on gene expression, as monomers, homodimers or heterodimers with RXR. TRs could also mediate indirect actions by interfering with other NRs signaling pathways to regulate common target genes [138]. Indeed, TH signaling, especially in metabolic regulation, involves TR crosstalk with other nuclear hormone receptors including PPARα, PPARγ and LXR [138]. Such crosstalk has not only been demonstrated in vitro but also in vivo, using mouse models. They could impact different molecular levels of gene regulation (Figure 2) [139,140].

### 5.1. TR Peripheral Crosstalk in Regulating Metabolism

Although, NRs interaction remains a complex mechanism that requires further investigation, it could be explained at least by the structural similarity in the DNA and ligand binding domains among NRs. All TR, PPAR and LXR are ligand-activated NRs that share similar structure and mode of action by binding to DNA response elements to form heterodimers with RXR [96]. First, the DNA binding domains are highly conserved among these NRs, containing two zinc fingers and arranged as direct repeats of hexameric half-sites, although the spacing of the hexamers varies among the different receptors. Second, NRs also share a very conserved leucine zipper, which is the interface for RXR heterodimerization [96]. Thus, NRs may compete for limiting amounts of RXR. Such a competition for RXR influences gene expression [141]. Moreover, TRs bind to TH with a higher affinity than PPAR and LXR bind to their natural ligands, conferring to TR a dominance in its interaction with RXR, and reflected by a greater effect on coregulated genes [138]. Third, this competition could be extended to common coactivators and corepressors. Indeed, the ligand binding domain (LBD), apart its ligand sequences specific for each receptor, contains regions interacting with other receptors as well as coactivators and corepressors [138].

Crosstalk between TR, PPAR and LXR has been reported, especially on cholesterol, lipid and glucose metabolism-related genes. Previous in vitro studies have underlined interactions between PPAR signaling and TR-dependent pathways [142]. Crosstalk between TR and PPAR signals could involve competition for their common heterodimeric-partner (RXR) [143], as well as for their respective responsive elements in target gene promoters. Indeed, TR has been shown to bind PPRE [144,145]. Nevertheless, the response to PPAR agonists may depend on the interactions between PPARs and TRs. In most cases, non-ligand binding TR mutant represses PPAR transcriptional activity [139].

Both TR and PPAR regulate expression of key enzymes of the fatty acid oxidation carnitine palmitoyl transferase Ia (CPT-Ia) and acyl–CoA oxidase (ACO). Crosstalk between TR and PPARα has been observed in regulation of *CPT-Ia* and *ACO*, especially in a mouse model of TRα (P398H) mutant [139]. This TRα mutant isoform is still able to bind to PPRE, thereby inhibiting PPARα-induced enzyme expression and, causing fatty acid accumulation in the livers of these mice [139]. Furthermore, lipoprotein lipase is regulated by both PPARγ. It was demonstrated that the mutant TRβ PV represses the PPARγ induced lipoprotein lipase gene expression, by binding to PPRE and by recruiting corepressors [137].

The role of LXR as a coordinator of both lipid and carbohydrate metabolism suggests the potential for interactions with TR. ATP-binding cassette transporter A1 (ABCA1) is a transporter of cellular non-esterified cholesterol and phospholipids in the liver, mainly regulated by LXR. TR competes with LXR for binding to the LXRE and inhibiting LXR-mediated *Abca1* gene expression [146,147]. Such examples of crosstalk and interaction are also observed in carbohydrate metabolism. A recent study describes that carbohydrate-response element-binding protein (ChREBP), a major transcription factor controlling the activation of glucose-induced lipogenesis in the liver, was characterized as a direct target of TRβ and LXR regulated in a tissue-selective manner. Both in vivo and in vitro assays showed a crosstalk between LXR and TRβ signaling on the *ChREBP* promoter, especially in the liver [148,149].

Furthermore, recent investigations support a complex interaction between LXR and PPARα in the regulation of glucose and lipid metabolism. The two NRs may either cooperate or have opposite effects on gene expression [150,151]. Yet the exact mechanisms underlying such crosstalk remain to be determined.

### 5.2. TR Crosstalk in the Regulation of Central Metabolism

Recent studies have demonstrated the expression of NRs within different brain regions, in particular at the hypothalamic level, the integrator of whole body energy homeostasis [77]. However, little is known about their role in the central control of energy homeostasis. Increasing data show interactions between TR and PPAR or LXR in the peripheral tissues (described above). Besides, such crosstalk at the central level remains less investigated. In this context, several studies focused on hypothalamic interactions between the different signaling pathways controlling metabolism.

Recent evidence supports a potential role for PPARs in the central energy homeostasis regulation [152]. In the CNS, all PPAR subtypes are expressed at different levels, however their function in the brain is not well elucidated. Of the three, PPARγ signaling pathway is the best characterized in the brain. Indeed, PPARγ and its cognate agonists appear to be attractive therapeutic targets for various disorders of the central nervous system [153,154]. PPARγ agonists have shown promising results in animal models of Alzheimer’s disease, stroke, multiple sclerosis, Parkinson’s disease, amyotrophic lateral sclerosis, and pituitary adenoma [155]. Further, it was confirmed that PPARγ isoform is expressed in key neuronal subsets regulating energy homeostasis [156]. More importantly, a recent study has demonstrated that mice lacking PPARγ in POMC neurons displayed increased energy expenditure and decreased fat mass and food intake. The absence of PPARγ was also associated with improved glucose metabolism and increased insulin sensitivity. Peripheral administration of either a PPARγ activator or inhibitor failed to affect food intake of mice with POMC-specific *PPARγ* ablation [124]. Besides, PPARα showed also an important effect on the brain. Intra-cerebroventricular (icv) and Intra-hypothalamic administration of the PPARα activator Wy14643 reduced glucose utilization and increased food intake in wild-type but not in *PPARα*-deficient mice [157]. Importantly, a previous report showed that the peripheral activation of PPARγ by rosiglitazone treatment affects hypothalamic-pituitary-thyroid axis and thyroid hormone release [143]. Taken together, these results suggest a pivotal role of hypothalamic PPARγ signaling pathway on central metabolic homeostasis, particularly on TH-dependent gene regulations.

Accordingly, an in vivo study tested the hypothesis of crosstalk between hypothalamic PPARγ and TRβ on T3-dependent regulation of the *Trh* promoter [158]. Our results showed first, that icv administration of PPARγ agonists leads to increased T3-independent *Trh* transcription and increased circulating T4 levels. In contrast, PPARγ antagonist did not affect *Trh* transcriptional activity in the absence of T3, but interfered with T3-dependent *Trh* repression. Then, silencing PPARγ protein levels by using small hairpin RNA (shRNA) increased T3-independent *Trh* transcription, whilst PPARγ overexpression abrogated T3-dependent *Trh* repression. Interestingly, the effect of PPARγ overexpression was reversed by co-expression of either TRβ1 or RXR [158]. These results suggest that PPARγ may interfere with TR signaling at the hypothalamic level, through a competition for limiting amounts of RXR.

LXRs are one class of nuclear receptors which are believed to be master integrators of cholesterol metabolism in the periphery [159]. Recently, their activation with a specific agonist has been shown to enhance cholesterol metabolism also in the CNS [160]. Furthermore, LXRs are expressed in the CNS, especially in the hypothalamus [161]. Thus, it suggests that LXR could play physiological and metabolic functions in the brain. Indeed, several arguments are in favor of this hypothesis, particularly via a crosstalk between the LXR and TR signaling pathways.

A recent study has revealed a crosstalk between TR and LXR in the regulation of Selective Alzheimer’s disease (AD) indicator-1 (*Seladin-1*) gene expression in an AD mouse model. Overexpressing Seladin-1 in the neurons increases the amount of cholesterol and avoids β-amyloid accumulation, oxidative stress and neurons apoptosis [162]. Both NRs have been shown to be involved in Seladin-1 gene expression, TR-β and LXR-α competitively up-regulating the human Seladin-1 promoter [140]. These results suggest that TR and LXR would co-regulate lipid metabolism in CNS. Interestingly LXR could be an attractive therapeutic target for neurodegenerative diseases [163,164].

Also, a crosstalk between TR and LXR has been recently reported, in the context of the hypothalamic TH negative feedback loop regulation: key target genes involved in the central control of metabolism, *Trh* and *Mc4r*, were impacted by this crosstalk between TR and LXR [135]. Indeed, using in vivo gene transfer, we explored the involvement of LXR in the hypothalamic metabolic pathways, analyzing the interference of LXR with the transcriptional regulation induced by TRs. Our results showed that activation of LXR by its specific agonist GW3965 repressed the transcriptional activity of both *Trh* and *Mc4r* promoters, and this only occurred in euthyroid mice. This repression was restored by TH treatment in hypothyroid mice, yet only in the *Trh* promoter [135]. Conversely, LXR knocked-down abrogated this repression, leading to a relative activation of the *Trh* promoter in the PVN. Further, in vivo ChIP results, showed that LXR was recruited to the *Trh* promoter region only in the presence of T3. Yet, no simultaneous recruitment of RXR and LXR on the *Trh* promoter region were observed [135]. Nevertheless, *LXR* KO mice showed enhanced secretion of TSH, thereby stimulating THs levels. Collectively, these results provide evidence that depletion of LXRs would abrogate the TH negative feedback loop in the hypothalamus and cause a loss of TRs in the PVN area [136,137].

Furthermore, the T3 signaling pathway could affect LXR transcriptional regulation. Indeed, qPCR results showed that T3 treatment of newborn mice induced the hypothalamic regulation of a number of LXR target genes implicated in metabolism and inflammation. Interestingly, key genes of inflammation, *Pparα*, *Tnfα* and *Il1* showed significant hypothalamic mRNA levels increase afterT3 treatment [135]. Thus, as in the periphery, the later genes would be LXR/TR targets in the hypothalamus. These results suggest that crosstalk between LXR and TR may be involved in the central regulation of inflammation. Since LXR activation by its specific ligand showed anti-inflammatory effects [165,166,167], this property could be exploited also in the CNS.

## 6. Conclusions

It is well demonstrated that TH are endocrine messengers with a profound impact on energy expenditure and appetite regulation. Accumulating evidences obtained from genetic mouse models and pharmacologic approaches pinpoint the TH signaling pathway as a master driver of metabolism regulation by acting, to a large extent, at the central level. Several studies have elegantly elucidated molecular mechanisms of action of TH in the brain. A continuous interaction between TH and key regulatory mechanisms coexist in the hypothalamus for a tightly controlled body weight and optimal energy balance. Remarkably, effects of THs are interrelated with key energy sensors in the brain. In addition, TH-mediated action is absolutely dependent upon its cognate receptors TRs that directly bind to target genes. Interestingly, TRs isoforms interact with other nuclear receptors that play a key role in metabolic regulation such as PPAR and LXR. Thus a deeper understanding of the mechanisms and interactions of TH signaling pathways in the hypothalamic control of metabolism will lead to identifying biomarkers and effective and selective targets that will improve the therapy of energy balance disorders, such as obesity.
